# On the use of fractional calculus to improve the pulse arrival time (PAT) detection when using photoplethysmography (PPG) and electrocardiography (ECG) signals

**DOI:** 10.1371/journal.pone.0298354

**Published:** 2024-02-16

**Authors:** Mahtab Mohammadpoor Faskhodi, Miguel A. Garcia-Gonzalez, Mireya Fernandez-Chimeno, Federico Guede-Fernández, Marc Mateu-Mateus, Lluis Capdevila, Juan J. Ramos-Castro

**Affiliations:** 1 Department of Electronic Engineering, Universitat Politècnica de Catalunya, Barcelona, Spain; 2 Value for Health CoLAB, Lisbon, Portugal; 3 Laboratory of Sport Psychology, Department of Basic Psychology, Universitat Autònoma de Barcelona (UAB), Bellaterra, Spain; 4 Sport Research Institute, Universitat Autònoma de Barcelona (UAB), Bellaterra, Spain; University of Oxford, UNITED KINGDOM

## Abstract

The pulse arrival time (*PAT*) has been considered a surrogate measure for pulse wave velocity (*PWV*), although some studies have noted that this parameter is not accurate enough. Moreover, the inter-beat interval (*IBI*) time series obtained from successive pulse wave arrivals can be employed as a surrogate measure of the *RR* time series avoiding the use of electrocardiogram (ECG) signals. Pulse arrival detection is a procedure needed for both *PAT* and *IBI* measurements and depends on the proper fiducial points chosen. In this paper, a new set of fiducial points that can be tailored using several optimization criteria is proposed to improve the detection of successive pulse arrivals. This set is based on the location of local maxima and minima in the systolic rise of the pulse wave after fractional differintegration of the signal. Several optimization criteria have been proposed and applied to high-quality recordings of a database with subjects who were breathing at different rates while sitting or standing. When a proper fractional differintegration order is selected by using the *RR* time series as a reference, the agreement between the obtained *IBI* and *RR* is better than that for other state-of-the-art fiducial points. This work tested seven different traditional fiducial points. For the agreement analysis, the median standard deviation of the difference between the *IBI* and *RR* time series is 5.72 ms for the proposed fiducial point versus 6.20 ms for the best-performing traditional fiducial point, although it can reach as high as 9.93 ms for another traditional fiducial point. Other optimization criteria aim to reduce the standard deviation of the *PAT* (7.21 ms using the proposed fiducial point versus 8.22 ms to 15.4 ms for the best- and worst-performing traditional fiducial points) or to minimize the standard deviation of the *PAT* attributable to breathing (3.44 ms using the proposed fiducial point versus 4.40 ms to 5.12 ms for best- and worst-performing traditional fiducial points). The use of these fiducial points may help to better quantify the beat-to-beat *PAT* variability and *IBI* time series.

## Introduction

The pulse arrival time (*PAT*) is becoming a useful measure of the cardiovascular system [1, 2] due to its convenient and noninvasive nature. *PAT*, for one cardiac cycle, is defined as the interval between the apex of the R wave measured using an electrocardiogram lead and the arrival of the pulse wave (pulse arrival (*PA*)) at a distal site, which is generally measured by plethysmographic methods (for example, using a photoplethysmographic pulse sensor attached to the wrist, finger, forehead, or ear). The *PAT* is used as a surrogate measure of the pulse wave transit time (*PTT*) which is defined as the time that the pulse wave elapses to travel between two locations, one downstream from the other [1]. If the location proximal to the heart is the aortic valve and the distal location is the same as that used to evaluate the *PAT*, both intervals can be related by:

PAT(n)=PTT(n)+PEP(n)−QR=PTT(n)+IVC(n)+EMD−QR (1)

The pre-ejection period (*PEP(n)*) is clinically defined from the start of the Q wave to the opening of the aortic valve [3] of the *n*^*th*^ measured cardiac cycle. Electromechanical delay (*EMD*) is defined from the start of ventricular depolarization (Q wave) to the beginning of the increase in pressure in the left ventricle and healthy subjects are in the range between 30 ms and 40 ms [4]. The *EMD* can be considered constant for the same individual so we assume that it does not change on a beat-to-beat basis [5]. Moreover, the QR interval (derived from the QRS complex) can also be considered a constant for the same individual during normal measuring intervals [6]. On the other hand, the isovolumetric contraction (*IVC*) interval corresponds to the increase in pressure in the left ventricle and ends with the opening of the aortic valve. This interval is regulated by the autonomic nervous system (ANS) [7]; hence, it has beat-to-beat variability. In summary, the beat-to-beat difference between *PAT* and *PTT* can be attributed to beat-to-beat changes in the *PEP* that are attributable to changes in the *IVC*. Kortekaas MC et al. [8] measured the contribution of the variability in the *PEP* to the variability of *PAT* and concluded that it was approximately 20% of the total variability.

The *PEP* increases when changing posture from supine to standing [5, 6, 9], decreases with physical activity [6, 10], and does not depend on its average value concerning respiratory frequency [9]. The *PEP* is highly modulated by mental and physical load [10]. The *PEP* is regarded as an indicator of sympathetic beta-adrenergic control of the heart period but only in stationary conditions, such as when posture does not change during the measurement [9]. If posture remains constant, an increase in the *PEP* can be interpreted as an increase in sympathetic beta-adrenergic activity. Because ventricular contractility and, accordingly, *PEP* is regulated by the sympathetic arch, it is reasonable to think that beat-to-beat changes in the *PEP* can be modeled as a low-frequency (LF) process with relevant spectral components under 0.1 Hz. Miyano H et al. [11] showed that in isolated dog hearts, the transfer function of changes in ventricular contractility induced by sympathetic stimulation at frequencies up to 5 Hz behaves as a lowpass system with decreasing magnitudes from frequencies as small as 0.02 Hz. Hence, it is expected that beat-to-beat changes in the *PEP* (when no other sources of uncertainty in its measurement are present) will smooth.

On the other hand, *PTT* is commonly used as a noninvasive method for estimating blood pressure and arterial stiffness. Several studies, such as [12 and 13], have stressed that *PTT* (or pulse wave velocity (*PWV*)) is more strongly correlated with systolic, mean, and diastolic blood pressure than is *PAT*. Hence, predictions of blood pressure from *PTT* measurements are more accurate than from *PAT* measurements. The cause of this lack of correlation using *PAT* is the aforementioned variability in *PEP*. Moreover, some studies have indicated that *PTT* and the *PEP* are not independent [13]. Despite all these complex relationships among cardiac intervals, some studies have recognized that the *PAT* could be employed as a reasonable surrogate for blood pressure estimation [14]. The lack of consensus among studies can be attributed not only to the physiological variabilities of *PEP* and *PTT* but also to inaccuracies in the measurement of the different cardiac intervals.

Although the R wave of an electrocardiogram (ECG) is a sharp and well-defined fiducial point, the pulse arrival at a distal site is not so well-defined. Hence, we can model the *PAT* of the *n*^*th*^ beat in a recording as follows:

$$PAT(n)=PA_{ideal}(n)+v_{PA}(n)-R(n)$$ (2)

where *PA*_*ideal*_*(n)* is the detected pulse arrival at the distal site using an ideal fiducial point detection algorithm that can change on a beat-to-beat basis due to physiological modulations of the pulse wave velocity and *PEP*. In (2), *ν*_*PA*_*(n)* is a stochastic process associated with the inaccuracy of real fiducial point detection and *R(n)* is the location of the R wave (we suppose that the time location uncertainty in the detection of the R wave is negligible before the detection of pulse arrival).

On the other hand, *PTT* and the *PEP* can be written as follows:

$$PTT(n)=PA_{ideal\;}(n)+v_{PA}(n)-Ao_{ideal}(n)-v_{Ao}(n)$$ (3)

$$PEP(n)=Ao_{ideal}(n)+v_{Ao}(n)-(R(n)-QR)$$ (4)

where *Ao*_*ideal*_*(n)* corresponds to an ideal fiducial point detection of the aortic valve opening and includes feasible physiological beat-to-beat modulations; *ν*_*Ao*_*(n)* is the inaccuracy modeling process associated with a real aortic valve opening detector, and QR is supposed to be constant. On the other hand, if the *PTT* is measured using the pulse wave at two sites (proximal and distal), which is the common way to assess *PWV* due to the difficulty of measuring aortic valve opening, then the *PTT* is defined as follows:

$$PTT_{PD}(n)=PA_{ideal\;Distal\;}(n)+v_{PA_{Distal}}(n)-PA_{ideal\;proximal}(n)-v_{PA_{proximal}}(n)$$ (5)

Eqs (1) to (5) stress that all the different intervals change from beat to beat. While measuring these intervals, what we want to retain are changes in physiological origin and are not associated with beat-to-beat inaccuracies in fiducial point determination. *PAT* and/or *PTT* variability analysis [15, 16, 17] is still an emergent field for cardiovascular control research, probably because of the low variance of the obtained time series (when measured without inducing physiological changes). The low variance of the signal dictates that inaccuracies in fiducial point detection should be very low to ensure a fair signal-to-noise ratio. Identifying a sufficiently accurate fiducial point could provide better estimations of beat-to-beat *PAT* and *PTT* time series, hence improving the ability of these analyses to gain better noninvasive knowledge of cardiovascular regulation.

On the other hand, the inter-beat interval (*IBI*), defined as the time series obtained by measuring the interval between consecutive *PA*s, has been proposed as a surrogate measure of the *RR* time series obtained from the successive intervals between consecutive R waves measured using ECG [18, 19]. As identified in some studies, fiducial point selection for pulse arrival detection has an impact on the agreement between the *IBI* and *RR*. We can write:

IBI(n)=PA(n+1)−PA(n)=R(n+1)+PAT(n+1)−R(n)−PAT(n)=RR(n)+PAT(n+1)−PAT(n) (6)

Hence, the differences between the two-time series that quantify their agreement depend on the accuracy of the detection of successive pulse arrivals.

Regardless of the application (*PAT* or *PTT* variability analysis, *RR* vs. *IBI* comparisons), fiducial point detection is a key factor that undermines the accuracy of measurements. Chiu YC et al. [20] compared several of the most commonly employed fiducial points for *PA* detection for pulse wave velocity (*PWV*) measurements: the foot of the pulse wave, the point with the maximum first derivative, the point with the maximum second derivative, and the point where the tangent lines through the foot and maximum derivative points intercept. This same study did not consider the peak of the pulse wave due to its sensitivity to the presence of wave reflections although the peak has been considered the principal or auxiliary fiducial point in many other studies [18], [21, 22, 23]. Previously we concluded [24] that for *PWV* determination, the best fiducial points are the tangent method [25] and the point at the maximum of the second derivative. Several other studies, such as [25], have recognized the impact of noise on fiducial point stability. Solà J et al. [26] proposed a parametric estimation of the pulse wave to obtain a fiducial point for each pulse wave arrival. This study revealed that the best fiducial points that provide a *PAT* with the greatest repeatability (i.e., providing a *PAT* time series with the lowest variability) are the fiducial points obtained from fitting the rising edge of the pulse to a hyperbolic tangent model (TANH), the maximum of the second derivative and the tangent method. In a previous work [24], we found that to maximize the agreement between the *RR* and the *IBI* time series obtained from a smartphone camera, the best fiducial point, among the foot, the maximum of the second derivative, and the maximum of the first derivative, was the last fiducial point when proper filtering of the photoplethysmogram (PPG) was performed. Hence, the choice of fiducial point detection depends on the intended application of the *PAT* measurement.

The set of fiducial points available in the previously mentioned (and other) studies is limited: a fiducial point is obtained by looking for a local maximum (or minimum) in the PPG after some processing or filtering has been performed. The resulting post-processed signal can be the difference between two fitted straight lines (tangent method), the peak or trough of a bandpass filtered version of the acquired PPG, the maximum of the first or second derivative of the filtered PPG, or the minimum of an error function between the filtered PPG and a fitting model (TANH method). Accordingly, previous research and the current study recognize the importance of accurate fiducial point detection for obtaining reliable *PAT* and *PTT* measurements. This study attempts to present a comprehensive set of fiducial points for *PAT* detection, optimize the accuracy of these selected fiducial points, and systematically compare these points with previously established points. Specifically, this study addresses specific challenges, such as the unpredictability of *PEP* variations (which leads to an increase in complexity when considering the interaction between *PEP* and *IVC* [8]) and the unclear nature of pulse arrival, and emphasizes practical applications, such as *PAT* variability analysis and *RR* vs. *IBI* comparisons. Furthermore, a comparison between *PAT* and *PWV* indicates that *PWV* has a stronger correlation with blood pressure, suggesting that *PAT* has limitations related to *PEP* fluctuation and the complex interaction between *PEP* and *PTT* [27]. Identifying accurate fiducial points is another challenge since the arrival of the pulse is not as clear as the distinct R wave seen in electrocardiograms. Consequently, the proposed model emphasizes the necessity of distinguishing ideal and actual detection methods, acknowledging the uncertain nature of real fiducial point detection. The need for precise detection to improve the accuracy of *PAT* and *PTT* measures in cardiovascular research and monitoring is highlighted by the crucial role that fiducial points play in measurements, as has been studied in several previous studies [24–26]. As a result, this study aimed to design a new approach for *PAT* detection, to address these challenges and improve the reliability of cardiovascular assessments. This study emphasizes the effectiveness of our proposed set of fiducial points in optimizing key objective criteria. Specifically, our findings show the potential to minimize agreement between the *RR* and *IBI* time series, as well as minimize variance in the *PAT* time series. Nevertheless, further investigations, possibly involving more extensive and potentially invasive studies, are imperative to determine the most suitable criterion for maximizing agreement between *PAT* and crucial cardiovascular parameters such as blood pressure or arterial stiffness.

## Materials and methods

In this section, we introduce a comprehensive set of fiducial points derived from identifying local maxima and minima following the fractional differintegration of the pulse wave. These points are then applied to high-quality recordings from a database, monitoring subjects exhibiting various breathing patterns. Subsequently, we determine the optimal fractional order for each recording using diverse optimization criteria. We then compare the performances of these optimal orders with those of other reference points related to pulse arrival detection that have been documented in the literature.

### Fiducial point based on fractional differintegration set proposal

Because the peak and foot of the pulse, the maximum of the first differentiation and the maximum of the second differentiation are commonly used fiducial points, a natural extension is to use the locations of local maxima and minima after computing the fractional differintegration of the systolic phase of the pulse wave for a large enough number of fractional orders as candidate fiducial points. The proposed fiducial points for pulse arrival time (*PAT*) measurements, derived from local maxima and minima in the systolic rise after fractional difference integration, offer several advantages over traditional methods. These methods demonstrate dynamic adaptability to varying pulse wave morphologies, increased specificity in localizing key events, and an improved signal-to-noise ratio. These points contribute to boosted beat-to-beat stability, reducing dependency on resting conditions and displaying potential for real-time applications. These fiducial points were chosen because of their physiological relevance and robust detection capabilities, as they can capture significant hemodynamic events during the systolic phase. This approach can reduce noise effects and maintains consistency between beats by considering local features, which are essential for reliable beat-to-beat analyses.

Fractional differintegration is an extension of differential calculus that has found practical applications in several fields of science [28]. In [29], we used a fast algorithm to approximate the discrete fractional differintegration of a discrete signal that relies on the Grünwald–Letnikov approach, which approximates the fractional differintegration as described in [28] by:

$$D^{\alpha }x(n)≅f_{s}^{\alpha }\cdot \sum_{j=0}^{n}{\left(-1\right)}^{j}\cdot \frac{\Gamma (\alpha +1)}{\Gamma (j+1)\cdot \Gamma (\alpha -j+1)}\cdot x(n-j)$$ (7)

where *x(n)* is the *n*^*th*^ sample of the signal to be fractional differintegrated, *f*_*s*_ is the sampling frequency of the signal that is a scaling factor irrelevant for our analysis because we are interested in locating maxima and minima, Γ(*k*) is the gamma function evaluated at *k* ∈ℝ and *α* ∈ℝ is the fractional order.

For *α* = 1 the differentiation of the signal is obtained because Γ(*z*) = ∞ if -*z* ∈ℕ so,

$$\begin{array}{l} D^{1}x(n)≅f_{s}\cdot \sum_{j=0}^{n}{\left(-1\right)}^{j}\cdot \frac{\Gamma (2)}{\Gamma (j+1)\cdot \Gamma (2-j)}\cdot x(n-j)=f_{s}\cdot \sum_{j=0}^{n}{\left(-1\right)}^{j}\cdot \frac{1}{\text{j}!\cdot \Gamma (2-j)}\cdot x(n-j)\\ =f_{s}\cdot [x(n)-x(n-1)] \end{array}$$ (8)

For *α* = 2 the second differentiation of the signal is obtained as:

$$D^{2}x(n)≅f_{s}^{2}\cdot \sum_{j=0}^{n}{\left(-1\right)}^{j}\cdot \frac{2}{j!\cdot \Gamma (3-j)}\cdot x(n-j)=f_{s}^{2}\cdot [x(n)-2\cdot x(n-1)+x(n-2)]$$ (9)

For *α* = 0, the signal remains unchanged:

$$D^{0}x(n)≅f_{s}^{0}\cdot \sum_{j=0}^{n}{\left(-1\right)}^{j}\cdot \frac{\Gamma (1)}{\Gamma (j+1)\cdot \Gamma (1-j)}\cdot x(n-j)=x(n)$$ (10)

For *α* = -1, the cumulative sum divided by the sampling frequency (integration) of the signal is obtained:

$$D^{-1}x(n)≅f_{s}^{-1}\cdot \sum_{j=0}^{n}{\left(-1\right)}^{j}\cdot \frac{\Gamma (0)}{\Gamma (j+1)\cdot \Gamma (-j)}\cdot x(n-j)=f_{s}^{-1}\cdot \sum_{j=0}^{n}x(n-j)$$ (11)

This last expression uses Euler’s reflection formula, which states:

$$\Gamma (z-n)={\left(-1\right)}^{n+1}\cdot \frac{\Gamma (-z)\cdot \Gamma (1+z)}{\Gamma (n+1-z)}\;\forall n\in \mathbb{Z}$$ (12)

Hence, for *j = n*+1 and *z* = *n*+1 = *j*:

$$\Gamma (1)=1={\left(-1\right)}^{j}\cdot \frac{\Gamma (-j)\cdot \Gamma (1+j)}{\Gamma (0)}$$ (13)

For *α* ∉ ℤ, signals different from that those obtained from simple differentiation or cumulative sums are obtained. Decreasing the value of *α* provides smoother versions of the signal, while increasing the value of *α* enhances rapid changes in the signal.

Eq (7) has accuracy problems due to the exponential-like behavior of the gamma function. In this work, we use the approximation proposed in [30] to overcome that problem. The fractional differintegration can be approximated by a filter:

$$D^{\alpha }x(n)≅f_{s}^{\alpha }\cdot \sum_{j=0}^{n}c_{j}^{\alpha }\cdot x(n-j)$$ (14)

where the coefficients of the filter are recursively obtained as follows:

$$c_{0}^{\alpha }=1,\;c_{j}^{\alpha }=\left(1-\frac{1+\alpha }{j}\right)\cdot c_{j-1}^{\alpha }$$ (15)

**Fig 1:**
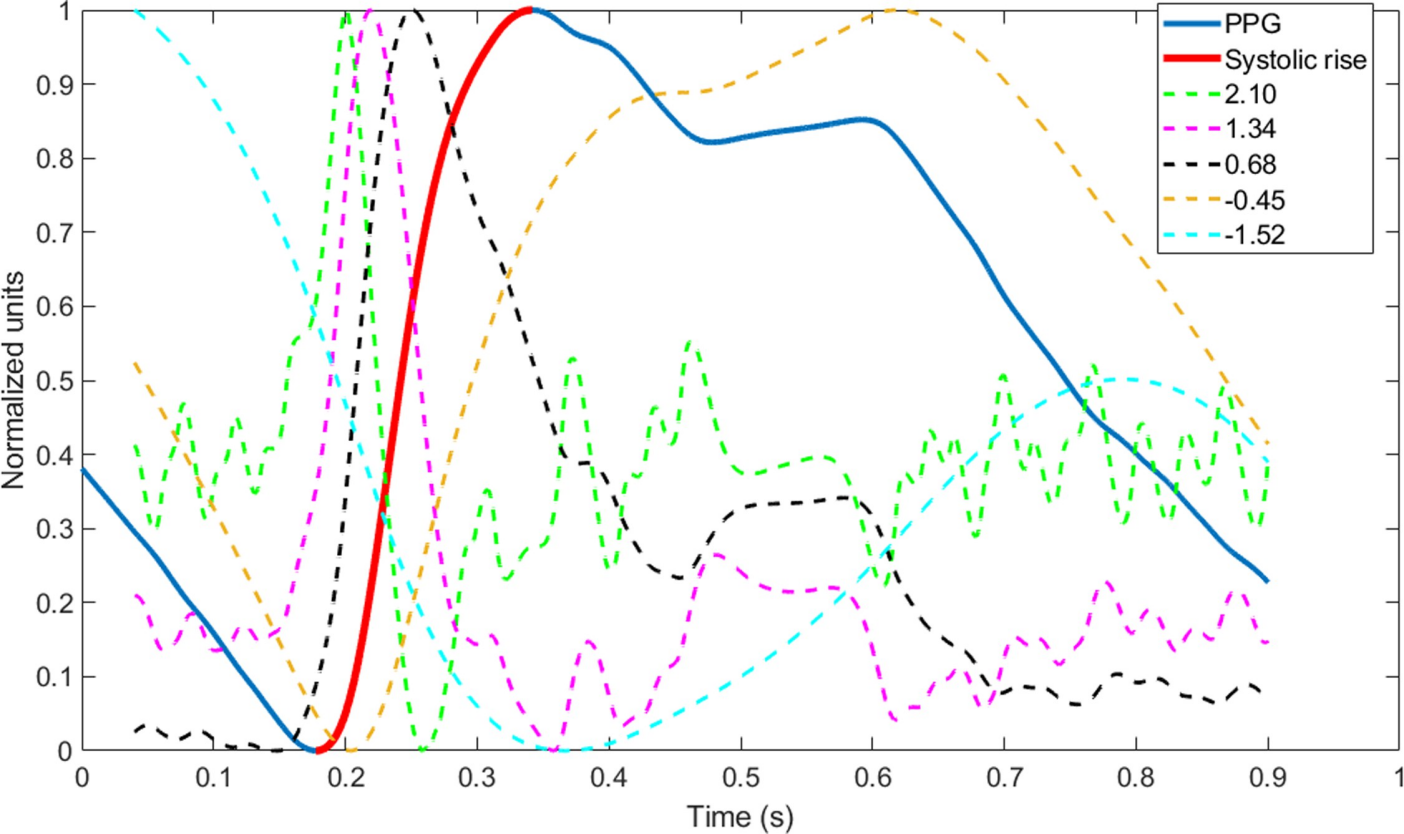
Example of fractional differintegrated versions of a pulse wave. The blue line shows a pulse wave encompassing around a cardiac cycle, while the red line corresponds to the systolic increase in the pulse wave. The dashed lines correspond to the application of fractional differintegrations to the signal using an arbitrary *α* (the values are shown in the legend). All signals were normalized between 0 (minimum) and 1 (maximum).

Fig 1 shows an example of a cycle of a pulse wave, its systolic rise, and some of its fractional differintegrated resulting signals. Note that by changing the value of *α*, the resulting signal may reach a maximum and/or a minimum during the systolic increase at different locations. We propose as an infinite set of candidate fiducial points for pulse arrival (*PA*) any maximum and minimum located inside the interval between the foot and peak of the pulse wave using a fractional differintegration of order *α*. In the following section, we will write $PA_{tag}^{\alpha }$(*n*) to denote the fiducial point obtained for the beat *n* using the fractional differintegrated of the order *α* signal and locate, when the *tag* is 1, its maximum inside the systolic rise or its minimum when the *tag* is -1. For example, $PA_{-1}^{0.57}$(38) is the minimum of the fractional differintegrated signal using a fractional order of 0.57 that was found during the systolic increase in the 38^th^ beat of the recording. Some optimization criteria based on the dynamics of $PA_{tag}^{\alpha }$(*n*) for all the available beats in the recording will allow us to optimize the values of *α* and the *tag*.

### Experiment description

This work aimed to compare the performance of several fiducial points for *PA* estimation by comparing time series that employ these fiducial points using recordings made under stationary and still conditions. We will compare our proposed set of fiducial points obtained from fractional differintegration of the pulse wave with other fiducial points employed in previous literature outlined in the Introduction section. We chose a subset of a series of previously recorded physiological signals that were the basis of the experiment described in [21] and added some recordings that were not used for that paper. The complete set of recordings is available at [31] in the Open Science Framework (OSF) repository.

#### Participants

The dataset comes from 20 adult volunteers without known cardiac or respiratory diseases (15 male, 5 female) aged 22 to 56 years (mean age: 32.3 years, standard deviation: 11.8 years). Their weight and height expressed as the mean ± standard deviation, were 73.9 kg ± 13.0 kg and 173 cm ± 9 cm, respectively. Written informed consent was obtained from all participants involved in the study. The study was conducted according to the guidelines of the Declaration of Helsinki and approved by the local Ethics Commission for Human Experimentation of the Universitat Autònoma de Barcelona (protocol code CEEAH-5745). The experiments began on October 20, 2021, and finished on February 15, 2022.

#### Data collection

For each subject, a session with a total of 9 recordings simultaneously sampling two ECG leads, PPG signals, and respiratory effort signals was carried out. Each recording lasted approximately 5 minutes. Six recordings corresponded to measurements while the subject was sitting, while the remaining three were made while the subject was standing. The position at the start of the session was chosen at random. The signals were collected at different breathing rates to assess the influence of breathing rate on the accuracy of the measurements. Respiratory sinus arrhythmia is related to the breathing rate [30].

In this study, we aimed to analyze, among other factors, the influence of this arrhythmia on the accuracy of heart rate variability (HRV) derived from the *IBI* and how different breathing rates may affect the *PA*. The breathing rates were 6 breaths per minute (bpm), 15 bpm, and free breathing (breathing at will). The 6 and 15 bpm breath frequencies were controlled using a visual aid presented to the subject via a smartphone application. While sitting, the subject breathed at 6 bpm during two recordings, at 15 bpm during another two recordings, and at will (free breathing) for the other two recordings. The order of the breathing rates was randomized. While standing, the subjects breathed in random order at 6 bpm, 15 bpm, and at will in each of the three recordings. After finishing the recordings for one posture, the subject changed actively to the other posture, and five minutes were allowed before the start of the remaining recordings. The details of the subjects, posture, and breathing rate for each recording are disclosed in [31]. For two subjects, one of the nine measurements was considered unsuitable for measurement due to the intermittent disappearance of the pulse wave, both of which corresponded to the standing position. Accordingly, a total of 178 recordings with a length of 5 min are available in the repository.

Each recording in the dataset contains four physiological signals: two leads of the ECG, the PPG, and the respiratory signal. The ECG was measured using 6 skin electrodes (3 M Red Dot 2560) placed in standard lead I and standard lead II and two ECG (Biopac SS2LB) cables. The pulse wave was measured with a PPG sensor (Biopac SS4LA) placed in the middle finger of the right hand. Moreover, the respiratory signal was measured with a respiratory effort transducer (Biopac SS5LB). All four channels were connected to a Biopac MP36E acquisition system, where the signals were properly conditioned and acquired using a sampling rate of 5 kHz. For the ECG, the bandwidth of the Biopac amplifier was set between 0.05 Hz and 150 Hz. A 2^nd^ order Notch filter at 50 Hz was also included to remove main interference. For the PPG acquisition, a hardware high pass filter with a cutoff frequency of 0.05 Hz and a software low pass filter with a cutoff frequency of 30 Hz were used. The respiratory signal was acquired using the preset configuration recommended for the SS5LB sensor.

### Data processing

In the data processing section, we detail the *PAT* and *IBI* measurements based on some optimization criteria. As a first step in the processing of the recordings, the standard lead I of the ECG was employed to detect the QRS waves using a custom QRS detector, which will be subsequently explained.

#### Beat detection in the ECG and PPG signals

The rough pulse arrivals (*PA*) in the PPG signal were also detected using a similar detector after proper filtering of the PPG. No ECG information was available for the pulse arrival detector, and no PPG information was available for the QRS detector.

The QRS detector is intended to be used in ECG signals with low disturbances such as noise, baseline drift, interference, or movement artifact while allowing the R wave amplitude to change due to breathing or other causes of QRS amplitude modulation. The ECG signals were first filtered using a bidirectional 4^th^ order Butterworth bandpass filter with cutoff frequencies between 10 Hz and 30 Hz. After filtering, a maximum filter was applied to the signal. The output of this filter for sample *n* is the maximum of the filtered ECG signal in the interval starting at n-W and finishing at n+W, where W is an input empirical parameter of the QRS detector. Most of the recordings provide accurate detection using W = 600 ms. The R peak locations correspond to the samples where the maximum filter output equals the value of the filtered ECG.

**Fig 2:**
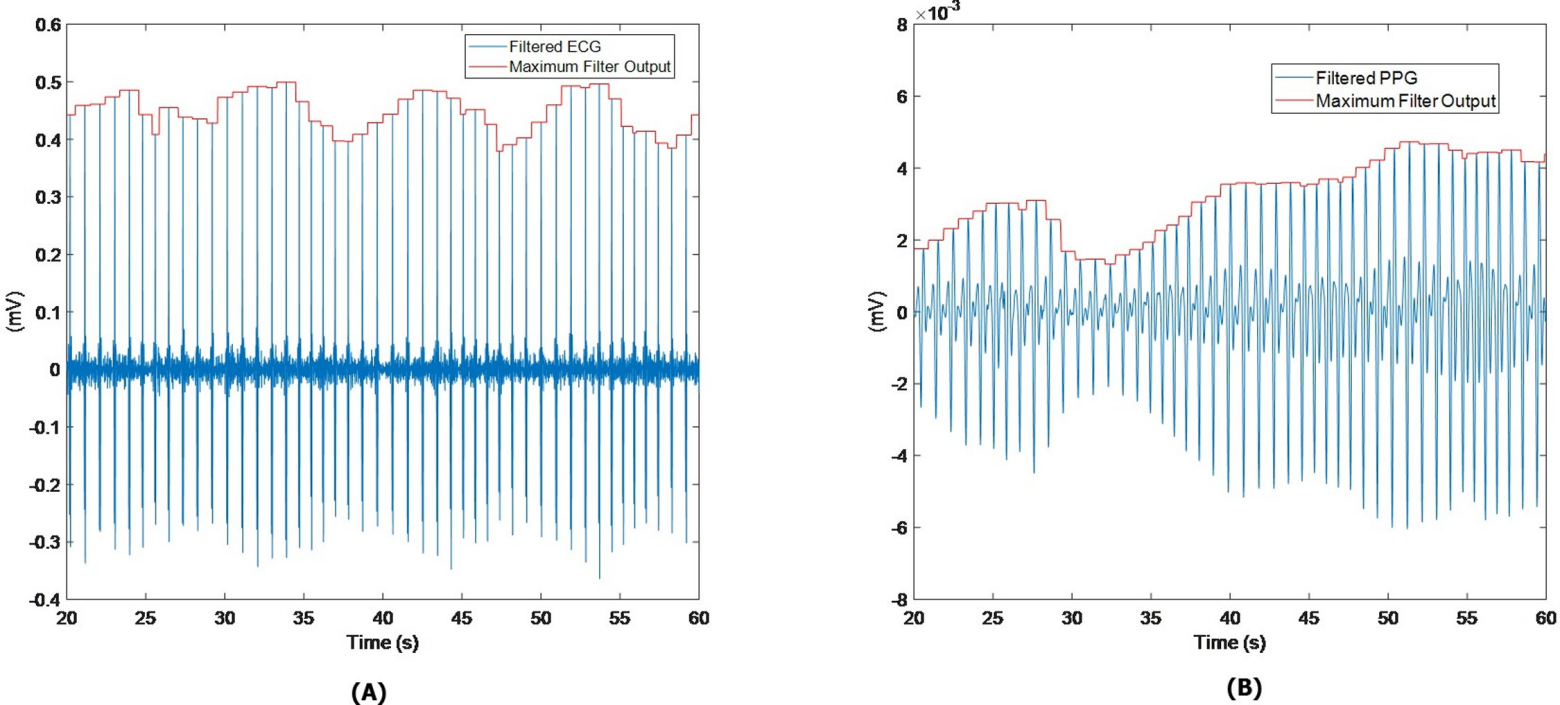
Example of QRS (panel A) and pulse arrival (panel B) detectors.

The pulse arrival detector for the PPG is quite similar. First, a bidirectional 4^th^ order Butterworth bandpass filter with empirically determined cutoff frequencies (between 2 Hz and 15 Hz enhances the systolic rise of the PPG for most morphologies) is applied. Next, a maximum filter with the same characteristics as the QRS detectors is used to locate the peaks of the filtered PPG. Fig 2 shows an example of both detectors.

All the MATLAB® codes for both detectors, including the implementation of the maximum filter, is available at [31]. R wave and rough pulse arrival detection were performed using these detectors while checking that the *RR* and *IBI* time series obtained from these detectors were similar to each other, from their physiological origin (by checking mean heart rate) and without (or with the smallest possible amount of) outliers. For a few ECG and PPG signals, the *RR* and *IBI* time series contained outliers when the detection was performed using the recommended W. For these recordings, the W parameter of the maximum filter was adjusted until the resulting time series was probed satisfactorily. For an even smaller set of *IBI* time series, the cutoff frequencies were also adjusted. At the end of this procedure, a set of R wave and pulse arrival (P) locations with very few outliers was obtained. These locations are also available for each recording in [31]. Although the R wave location will be considered the fiducial point for the ECG against which the *PA* will be compared, the rough P locations will be the starting point for the definition of new *PA* fiducial points in the next sections. No outlier correction was performed at the R or P locations because accuracy measures automatically reject them, as will be later explained. Nevertheless, if the first detected P location occurs before the first detected R wave, this P location is rejected. Moreover, if the last R wave does not have a P location after it, this R wave location is also rejected.

#### Fiducial point set generation

Once rough *PA* fiducial points are obtained for each recording, this section presents the general approach to optimizing the *PA* fiducial points based on fractional differintegration using three different optimization criteria. These criteria aim to find an optimal *α* and *tag* for each recording to minimize the standard deviation of a certain time series. As we will see, each criterion will be different from another because of this time series definition. The differences between the optimized *PA* fiducial points according to the different criteria and among other *PA* fiducial point definitions (i.e., using the TANH or the foot of the pulse wave) will also be assessed.

For each recording, the first and last R and P locations were removed for analysis to avoid searching for fiducial points before the start or after the end of the recording. Before the fractional differintegration computation using Eq (14) and (15), the PPG was first bandpass filtered using a 4^th^ order bidirectional Butterworth filter with cutoff frequencies set at 0.5 Hz and 15 Hz.

Next, the peak of each pulse wave was obtained by determining the maximum of the filtered PPG signal in an interval starting at the P rough location (*PA*_*rough*_) and ending 100 ms later. This peak will be one additional fiducial point for comparison of the results (*PA*_*peak*_). Next, the foot of each pulse wave was obtained (*PA*_*foot*_) by searching for the minimum of the filtered PPG in an interval starting 300 ms before the peak and ending at the peak location. The foot of each pulse will be another fiducial point for comparison with our proposed fiducial point. Once the foot and peak were located for each *PA* defining the start and end of the systolic rise, an interval starting 40 ms before the foot and ending at the peak was defined for each beat. This filtered PPG interval was fractional differintegrated using different *α* values, and the maximum or minimum location of the resulting signal inside the systolic rise was considered a new candidate fiducial point. We started the fractional differintegration 40 ms before the foot to mitigate the impulse response of the filter defined by Eqs (14) and (15). For each candidate fiducial point obtained using the same *α* and *tag*, a $PA_{tag}^{\alpha }$(*n*) time series was obtained. For each recording, we tested 4002 different candidate fiducial point time series using an *α* from -10 (integration of tenth order) to 10 (differentiation of sixth order) in steps of 0.01 and the two values of *tag* (-1 for the search of local minima and 1 for local maxima). Hence, this procedure started with a *PA*_*rough*_(*n*) time series and produced a 4002 $PA_{tag}^{\alpha }$(*n*) time series. Moreover, two additional time series for comparison (*PA*_*peak*_(*n*) and *PA*_*foot*_(*n*)) were obtained in intermediary steps. Nevertheless, if $PA_{1}^{0}$(*n*) = *PA*_*peak*_(*n*) and $PA_{-1}^{0}$(*n*) = *PA*_*foot*_(*n*), then they are included in the set of time series considered for optimization.

For the sake of comparison, the peak, foot, and starting P rough locations, the next fiducial point time series was also computed:

- The maximum of the first differentiated filtered PPG signal (*PA*_*pfd*_(*n*) but also included in the candidate fiducial point time series set as $PA_{1}^{1}$(*n*)).
- The maximum of the second differentiated filtered PPG signal (*PA*_*psd*_(*n*) was also included in the candidate fiducial point time series set as $PA_{1}^{2}$(*n*)).
- The average location of the TANH fitting of the filtered PPG (*PA*_*tanh*_(*n*)).
- The inception point of the tangent line at the maximum of the first differentiated filtered PPG with the foot level was also recorded (*PA*_*tan*_(*n*)).

The code for the computation of the different time series as well as the files containing the sets of the different fiducial points for each recording can be found in [31].

#### Optimization by maximizing the agreement of the *IBI* and *RR* time series

The first optimization consists of maximizing the agreement between the *RR* time series obtained from the successive intervals between R wave locations and the *IBI* time series obtained from the successive intervals between *PA* locations (*maxagr* optimization criterion). The beat-to-beat disagreement between both time series is defined as follows:

da(n)=IBI(n)−RR(n)=PA(n+1)−PA(n)−R(n+1)+R(n) (16)

Moreover,

da(n)=PA(n+1)−PA(n)−R(n+1)+R(n)=PAT(n+1)−PAT(n) (17)

Hence, the disagreement between time series can also be interpreted as the differentiation of the *PAT* time series.

The maximization of agreement employed in this work consists of choosing a proper pair of *α* and *tag* for the fractional differintegrated derived fiducial points that minimize the standard deviation of the disagreement time series:

$$\left(\alpha_{opt},\;tag_{opt\;}\right)=\arg\underset{\alpha ,\;tag}{\min}\sigma \left(PA_{tag\;}^{\alpha }(n+1)-PA_{tag\;}^{\alpha }(n)-R(n+1)+R(n)\right)$$ (18)

For the estimation of the standard deviation (*σ*) of the disagreement time series, samples of the *da*(*n*) time series with absolute values higher than 300 ms were not included. To gain further insight into the dynamics of the time series after fiducial point optimization, the maximum agreement (or minimum disagreement) time series is defined as follows:

$$mda(n)=PA_{tag_{opt}}^{\alpha_{opt}}(n+1)-PA_{tag_{opt}\;}^{\alpha_{opt}}(n)-R(n+1)+R(n)$$ (19)

and will be used for comparison to the corresponding time series obtained using other fiducial points. The *PAT* time series defined using $PA_{tag_{opt}}^{\alpha_{opt}}$(*n*)-R(*n*) will also be considered for comparison with the *PAT* time series obtained using other fiducial points.

#### Optimization by minimization of the variance of the *PAT* time series

If we suppose that the *v*_*PA*_(*n*) process in Eq (2) is uncorrelated with the pulse arrival time, the variance in the pulse arrival time and the variance in the inaccuracy process provide good estimates of the total variance in the *PAT* time series. Hence, minimizing the variance of the *PAT* time series equals minimizing the variance of *v*_*PA*_(*n*) if *PA(n)* and *v*_*PA*_(*n*) are independent (while the inaccuracies in R detection can be neglected). The second optimization criterion consists of minimizing the standard deviation of the *PAT* time series (*minSDPAT* optimization criterion):

$$\left(\alpha_{opt},\;tag_{opt}\right)=\arg\underset{\alpha ,\;tag}{\min}\sigma \left(PA_{tag}^{\alpha }(n)-R(n)\right)$$ (20)

For the estimation of the standard deviation (*σ*) of the *PAT*, those *PAT* samples with absolute values higher than 300 ms were not included. As with the previous criterion, the *mPAT* time series is defined as follows:

$$mPAT(n)=PA_{tag_{opt}}^{\alpha_{opt}}(n)-R(n)$$ (21)

will be used for comparison with those obtained using other fiducial points.

#### Optimization by minimizing the variance of the high pass filtered *PAT* time series

Under the hypothesis that *PTT* variability is regulated by changes in blood pressure and arterial stiffness and that both variables are regulated by the sympathetic nervous system, it is feasible to think that a *PAT* time series that mimics changes in *PTT* should have smooth dynamics. Hence, the minimization of the standard deviation of the *PAT* time series after high pass filtering using a 0.15 Hz cutoff frequency is proposed as a third optimization criterion (*minSDHPF* optimization criterion).

Because the *PAT* time series is unevenly sampled, to obtain the high pass filtered *PAT* time series (*HPFPAT*), the original *PAT* is resampled at 4 Hz using linear interpolation first obtaining an evenly sampled *PAT* time series (*esPAT*(*k*)). Those *PAT* intervals that were deemed outliers (those with a departure greater than 300 ms from the average *PAT*) were skipped from the interpolation procedure. Then, a bidirectional (linear phase) 4^th^ order high pass Butterworth filter, with a 0.15 Hz cutoff frequency is applied. Then, the applied optimization criterion is:

$$\left(\alpha_{opt},\;tag_{opt}\right)=\arg\underset{\alpha ,tag}{\min}\sigma \left(HPF\left(PA_{tag}^{\alpha }(n)-R(n)\right)\right)$$ (22)

where the operator *HPF ()* denotes the interpolation and filtering procedure previously described. Like in the previous criteria, the smooth, evenly sampled *PAT* time series is defined as follows:

$$sesPAT(k)=esPAT(k)-HPF\left(PA_{tag}^{\alpha_{opt}}(n)-R(n)\right)$$ (23)

and compared with the dynamics of the corresponding time series employing other fiducial points. The *PAT* time series defined using $PA_{tag_{opt}}^{\alpha_{opt}}(n)-R(n)$ will also be considered for comparison with the *PAT* time series obtained using other fiducial points.

The code for the estimation of $PA_{tag_{opt}}^{\alpha_{opt}}$ for the different optimization criteria as well as the files containing the sets of the different fiducial points for each recording can be found in [31].

#### Statistical analysis of α_opt_ and tag_opt_

After processing all the recordings and obtaining the different fiducial points, the first study among the *PA* fiducial points characterized the values of *α*_*opt*_ and *tag*_*opt*_ among the different optimization criteria. Because *α*_*opt*_ is not normally distributed but has a long-tailed median, 10% and 90% percentiles were obtained. Pairwise paired sign tests were used to assess the statistical significance of the difference in the median *α*_*opt*_ for the different optimization criteria. Because *tag*_*opt*_ can only have two possible values, it follows a Bernoulli distribution. The percentage of times that *tag*_*opt*_ is 1 (how many times the fiducial point corresponds to a maximum) is estimated for each optimization criterion. Once again, pairwise paired sign tests were used to assess the statistical significance of differences in *tag*_*opt*_ according to the optimization criteria. Moreover, unpaired sign tests for *α*_*opt*_ and *tag*_*opt*_ for each optimization criterion were performed to assess whether the values of*α*_*opt*_ and *tag*_*opt*_ showed statistically significant differences from zero median distributions. In the case of *α*_*opt*_, a statistically significant difference will show that the optimization criterion is more prone to obtaining fiducial points corresponding to fractional integrating or fractional differentiating the pulse wave; for *tag*_*opt*_, each optimization criterion will show if a maximum is more likely to be chosen instead of a minimum. In addition, nonparametric Fisher tests were employed to determine statistically significant nonrandom associations between the sign of *α*_*opt*_ and *tag*_*opt*_ for the different optimization criteria. Finally, regarding the relationships of the values of *α*_*opt*_ and *tag*_*opt*_ with several potential influential factors, the nonparametric Kruskal‒Wallis test was applied for each optimization criterion to ascertain whether their values are affected by inter-subject variability, breathing pattern, or posture.

#### Fiducial point comparison

To compare among the fiducial points, several features of the *PAT* time series obtained using $PA_{tag_{opt}}^{\alpha_{opt}}$ for the three optimizations and those obtained using the other seven fiducial points were obtained. The main reason for selecting these seven fiducial points lies in their prevalence as commonly identified optimal points in the PPG waveform, as reported in previous studies. Hemon and Phillips [32] compared methods for deriving instantaneous pulse periods (*PP*) from PPG signals, finding the best correlation with *RR* intervals using the intersecting tangent method. Also, several studies used high-frequency features, such as the foot, for *PAT* estimation, emphasizing their superiority over low-frequency fiducial points [33, 34]. Moreover, PPG signal derivatives are employed to enhance the recognition of points of interest and facilitate waveform interpretation [35, 36]. However, applying these fiducial points directly to noisy and corrupted PPG signals often results in unreliable and erratic results, compromising the repeatability of clinical *PAT* measurements. Hence, sophisticated methods for PPG waveform feature detection, including multi-dimensional parametric estimation [26] and the diastole-patching method [37] have been developed.

Previously, for feature estimation, outliers in each *PAT* time series and for each fiducial point were detected and corrected. To do so, a median filter with a window of 11 samples was applied to each time series. Any departure of the time series from the median filtered time series higher than 50 ms was considered an outlier. The selected features were estimated by skipping the detected outliers. The percentage of outliers concerning the total number of *PAT* intervals was annotated for further reporting.

The selected features were the mean value of the *PAT* ($\bar{PAT}$), the standard deviation of the *PAT* (*σ*_*PAT*_), and an approximation of the contribution of respiratory activity to *PAT* variability (*ResprPAT*). To derive a time series representative of respiratory activity synchronous with the *PAT* and having the same number of samples, the respiratory signal was sampled at each R wave location to obtain the *RsRESP* time series as follows:

RsRESP(n)=RESP(R(n)) (24)

where *RESP*(*k*) is the *k*^*th*^ sample of the acquired respiratory signal as previously described and *R(n)* is the sample at which the *n*^*th*^ beat out of *N* has been detected. For each analyzed *PAT* time series, the *ResprPAT* was estimated from the value of the cross-correlation function between the *RsRESP* and *PAT* time series evaluated at the delay that provides the maximum absolute value of cross-correlation as follows:

The cross-correlation function was estimated as,

$$\begin{array}{c} R_{PATRsRESP}(m)=\\ \left\{\begin{array}{cc} \sum_{n=0}^{N-m-1}(PAT(n+m)-\bar{PAT})\cdot (RSRESP(n)-\bar{RsRESP}) & m\geq 0\\ \sum_{n=0}^{N-m-1}(PAT(n)-\bar{PAT})\cdot (RsRESP(n-m)-\bar{RsRESP}) & m<0 \end{array}\right. \end{array}$$ (25)

The respiratory contribution to *PAT* was estimated as follows:

$$ResprPAT=|\frac{R_{PAT\;RsRESP}(k)}{N\cdot \sigma_{RsRESP}}|$$ (26)

where,

$$k=\arg\underset{m}{\max}|R_{PAT\;RsRESP}(m)|$$ (27)

and *σ*_*RsRESP*_ is the sample standard deviation of the *RsRESP* time series.

To compare among fiducial points, for each of the three features ($\bar{PAT}$,*σ*_*PAT*_ and *ResprPAT*) and each fiducial point, the 10%, 50% (median) and 90% percentiles were first obtained. While a comparison of $\bar{PAT}$ among fiducial points provides information on the central location of the fiducial points over the systolic rise of the pulse wave (being the foot and the peak fiducial points in their most extreme cases), a comparison of *σ*_*PAT*_ will reveal the contribution of each fiducial point to the total variability of the *PAT*. Finally, a comparison of the *ResprPAT* will reveal whether there are relevant differences in the contribution of respiratory parameters to *PAT* variability. To perform this comparison, pairwise paired sign tests were performed to assess significant differences in features between pairs of fiducial points.

To assess the influence of inter-subject variability, breathing pattern, and posture, the nonparametric Kruskal‒Wallis test was applied to each feature and fiducial point. The χ^2^ statistic and its corresponding significance were assessed to determine which fiducial points were most prone to influence by each of the three controlled factors.

For the ability of the different fiducial points to provide accurate measurements of the *RR* time series, the differences between the *IBI* time series defined as in Eq (6) and the *RR* time series defined as *RR(n) = R(n+1)* ‒*R(n)* were analyzed for each fiducial point. Agreement between both time series was quantified from the standard deviation of the difference between time series that equals the differentiation of the *PAT* time series (*PAT*(*n*+1)-*PAT*(*n*)), as shown in Eq (6). The standard deviation of the differentiated *PAT* time series (*σ*_*RR-IBI*_) was analyzed in the same way as the previous features were.

The p<0.001 was considered to indicate statistical significance, while p<0.05 was considered to indicate statistical significance.

## Results

Table 1 shows the median, 90%, and 10% percentiles of *α*_*opt*_ and the percentage of times that *tag*_opt_ is positive (indicating that the fiducial point corresponds to a maximum) according to the optimization criterion. Paired sign tests revealed that, compared with those of either the *maxagr* or *minSDHPF*, the *α*_*opt*_ and *tag*_opt_ of the *minSDPAT* were significantly different. Nevertheless, no significant differences were detected for *α*_*opt*_ or *tag*_opt_ when comparing *maxagr* and *minSDHPF*. According to unpaired sign tests, the values of *α*_*opt*_ showed a median very statistically significant difference of zero for *maxagr*, but the difference was not statistically significant for *α*_*opt*_ when using the *minSDPAT* or *minSDHPF* optimizations. On the other hand, the values of *tag*_opt_ show a highly statistically significant difference of zero for *maxagr* and *minSDHPF* but not for *minSDPAT*. A nonparametric Fisher test showed that there were very statistically significant nonrandom associations between the sign of *α*_*opt*_ and *tag*_*opt*_ (77.0% chance of having the same sign for *maxagr*, 82.6% for *minSDPAT* and 74.7% for *minSDHPF*).

**Table 1:** Descriptive statistics for *α*_*opt*_ and *tag* for each optimization criterion.

	Median(*α*_*opt*_)	Percentile 90% (*α*_*opt*_)	Percentile 10% (*α*_*opt*_)	Percentage *tag*_opt_ >0
*maxagr*	-0.68	2.03	-7.41	34.3%
*minSDPAT*	-0.12	2.90	-7.56	52.2%
*minSDHPF*	-0.48	7.70	-7.14	32.6%

Table 2 shows the χ^2^ statistics as the result of nonparametric Kruskal‒Wallis tests on the values of *α*_*opt*_ and *tag*_*opt*_ assessing the effect of the three controlled factors (subject, breathing rate, and posture) disclosed by the optimization criterion compared by the three controlled factors. The results showed that the values of *α*_*opt*_ and *tag*_*opt*_ are strongly dependent on the individual and the posture measured but are not associated with the breathing pattern. The median *α*_*opt*_ values for *maxagr* were -0.76 while sitting (N = 120) and -0.07 while standing (N = 58); for *minSDPAT*, the values were -0.38 and 1.73, respectively; and for *minSDHPF*, the values were -0.60 and 0.91, respectively. Hence, *α*_*opt*_ tends to be lower when sitting than when standing. The chance that *tag*_*opt*_ is equal to 1 for *maxagr* is 28.3% while sitting and 46.6% while standing; for *minSDPAT*, it is 45.8% and 65.5%, respectively; and for *minSDHPF*, it is 25.0% and 51.7%, respectively. Hence, the fiducial point is more likely to be at its maximum while standing than while sitting.

**Table 2:** Results of the Kruskal‒Wallis nonparametric test comparing the medians of variables for several groups. The table shows the χ^2^ statistic and its significance (n not significant, † p<0.05, ‡ p<0.001). The factors of subject, breathing and posture were tested separately.

***maxagr***		**Subject**	**Breathing**	**Posture**
	***α***_***opt***_	30.0^†^	1.72^n^	8.57^†^
	***tag***_***opt***_	43.7^‡^	1.32^n^	5.73^†^
***minSDPAT***		**Subject**	**Breathing**	**Posture**
	***α***_***opt***_	44.4^‡^	1.72^n^	10.2^†^
	***tag***_***opt***_	45.1^‡^	0.04^n^	6.04^†^
***minSDHPF***		**Subject**	**Breathing**	**Posture**
	***α***_***opt***_	42.5^‡^	0.78^n^	7.38^†^
	***tag***_***opt***_	34.5^†^	2.89^n^	14.3^‡^

The median percentage of outliers in the *PAT* time series was zero for all the fiducial points except for P_peak_ (0.31%), indicating that at least 50% of the recordings were free of any outliers for most fiducial points. The mean percentage of outliers was 0.67% for P_rough_, 1.32% for P_peak_, 1.21% for P_foot_, 0.35% for P_pfd_, 0.73% for P_psd_, 0.43% for P_tanh_, 0.33% for P_tan_, 0.25% for P_maxagr_, 0.23% for P_minSDPAT_ and 0.25% for P_minSDHFPAT_. The 95% percentiles for the percentages of outliers were 3.44% for P_rough_, 6.62% for P_peak_, 4.40% for P_foot_, 2.69% for P_pfd_, 1.87% for P_psd_, 1.40% for P_tanh_, 1.87% for P_tan_, 1.61% for P_maxagr_, 1.57% for P_minSDPAT_ and 1.61% for P_minSDHFPAT_.

**Fig 3:**
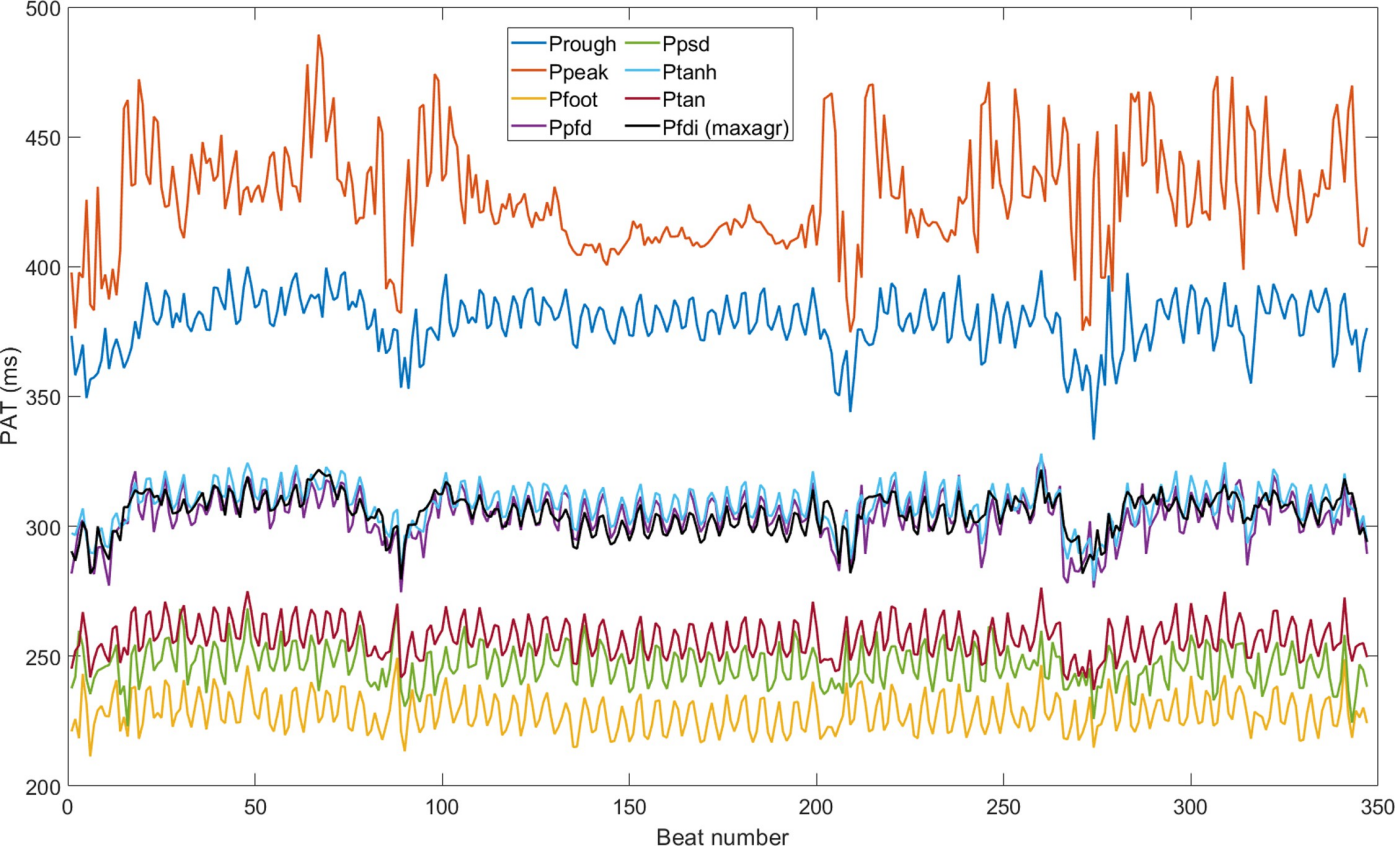
*PAT* time series obtained from different fiducial points defined during the systolic rise of the pulse wave. For the definition of the optimal fractional differintegration fiducial point, the *maxagr* optimization criterion was employed.

**Fig 4:**
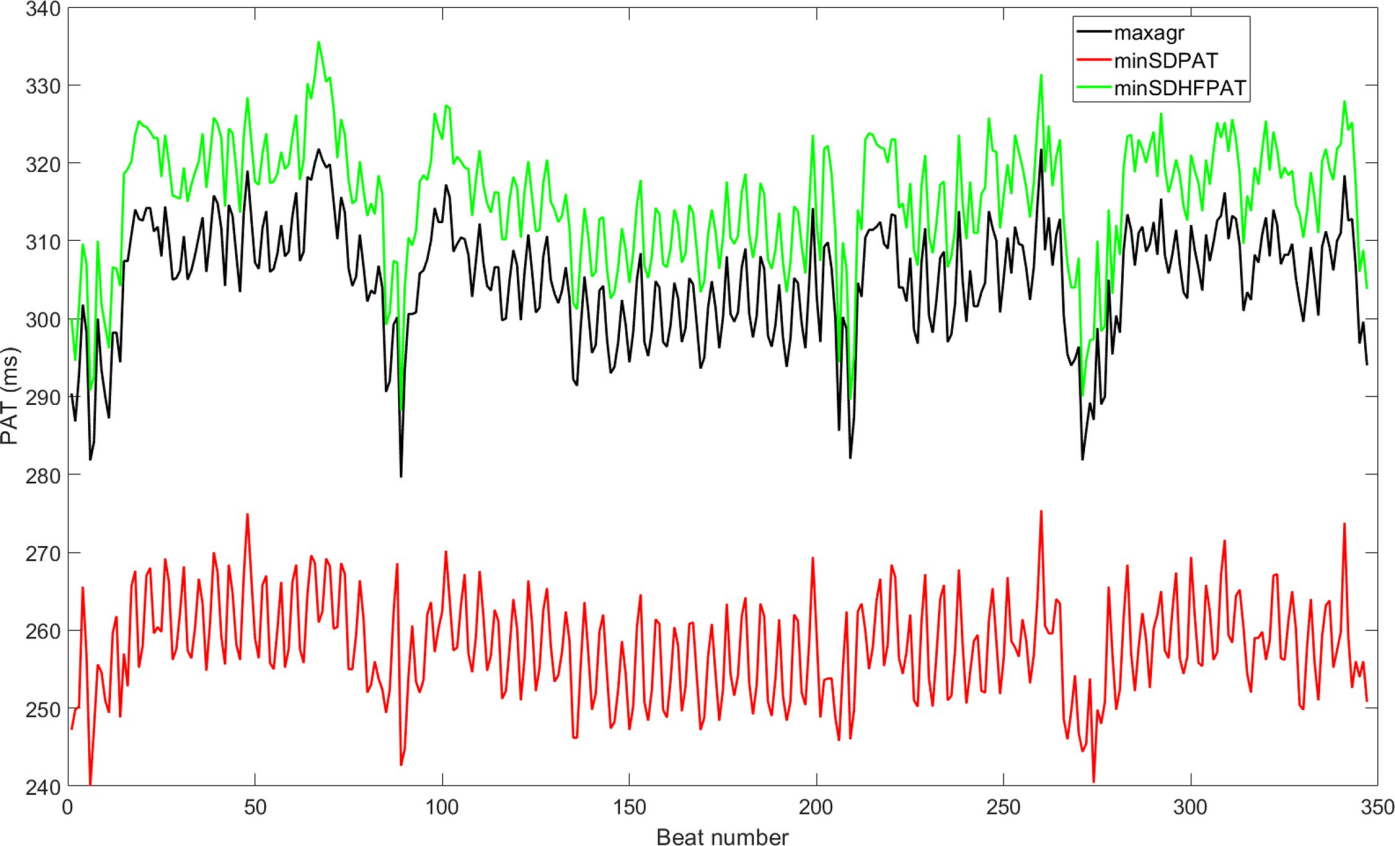
*PAT* time series for the same example shown in Fig 3. It is obtained from optimal fractional differintegration derived fiducial points using the three proposed optimization criteria.

Fig 3 shows an example of a measured *PAT* time series defined for different fiducial points, while Fig 4 compares the *PAT* time series using the optimal fiducial points obtained by fractional differintegration for the same recording of Fig 3 but using the three different optimization criteria. As shown in Figs 3 and 4, the mean *PAT* changes depending on the choice of fiducial point. Moreover, standard deviation and breathing activity could be influenced by fiducial point choice. For example, the mean *PAT* will reach a minimum when using the foot of the pulse wave and will reach a maximum when using the peak of the pulse wave, while the remaining fiducial points will have a mean *PAT* within. Fig 4 shows that the *PAT* with the lowest standard deviation using the optimal fractional differintegration fiducial point with the *minSDPAT* optimization criterion also had the most apparent contribution to breathing activity when compared with the other two criteria (the subject was breathing at 15 bpm in this particular example).

Table 3 shows the median, 10th percentile, and 90th percentile of the mean and standard deviation of the *PAT* time series as well as for the *ResprPAT* index and the standard deviation of the difference between the *RR* time series and the *IBI* time series. The results are disclosed by the fiducial point. Table 4 shows the z values of the paired sign tests for the features for each pair of fiducial points.

**Table 3:** Descriptive statistics for *PAT* time series characterization and agreement of *IBI* with *RR* time series disclosed by fiducial point. The values are the medians and the [10%– 90%] percentiles of the indices.

Fiducial point	$\bar{PAT}$ (ms)	*σ*_*PAT*_ (ms)	*ResprPAT* (ms)	*σ*_*RR-IBI*_ (ms)
P_rough_	371 [312–419]	10.2 [6.13–21.7]	5.12 [2.63–9.83]	7.97 [4.32–18.1]
P_peak_	407 [353–465]	15.4 [7.46–26.1]	4.56 [2.15–8.28]	9.93 [4.94–23.4]
P_foot_	231 [195–254]	8.22 [4.68–15–1]	4.72 [2.13–9.13]	7.29 [3.82–15.8]
P_pfd_	302 [262–335]	9.09 [5.08–18.1]	4.69 [2.27–8.84]	7.56 [3.58–14.5]
P_psd_	253 [221–286]	8.54 [4.72–15.4]	4.47 [2.15–8.67]	7.30 [3.79–15.0]
P_tanh_	306 [267–338]	8.67 [5.01–16.6]	4.50 [1.88–8.81]	6.62 [3.15–12.9]
P_tan_	260 [224–290]	7.66 [4.52–13.9]	4.40 [1.99–8.52]	6.29 [3.19–12.4]
P_maxagr_	294 [234–406]	7.80 [4.66–15.6]	3.58 [1.47–7.51]	5.72 [2.80–11.8]
P_minSDPAT_	270 [213–375]	7.21 [4.30–13.4]	3.79 [1.92–7.54]	6.29 [3.35–12.6]
P_minSDHFPAT_	307 [240–407]	8.16 [4.78–16.4]	3.44 [1.31–7.73]	5.80 [2.80–11.9]

**Table 4:** z value of the paired sign test comparing the feature for fiducial point A with the feature for fiducial point B. A positive z value means that the median of the feature for fiducial point A is larger than that for fiducial point B. Significance of the test is indicated as † for p<0.05 and ‡ for p<0.001.

*** $\bar{PAT}$ ***	A B	P_rough_	P_peak_	P_foot_	P_pfd_	P_psd_	P_tanh_	P_tan_	P_maxagr_	P_minSDPAT_
	P_peak_	-13^‡^								
	P_foot_	13^‡^	13^‡^							
	P_pfd_	13^‡^	13^‡^	-13^‡^						
	P_psd_	13^‡^	13^‡^	-13^‡^	13^‡^					
	P_tanh_	13^‡^	13^‡^	-13^‡^	-13^‡^	-13^‡^				
	P_tan_	13^‡^	13^‡^	-13^‡^	13^‡^	-12^‡^	13^‡^			
	P_maxagr_	7.7^‡^	13^‡^	-13^‡^	0.7	-9.2^‡^	2.0^†^	-8.0^‡^		
	P_minSDPAT_	11^‡^	13^‡^	-13^‡^	6.1^‡^	-2.0^†^	6.2^‡^	-0.8	7.6^‡^	
	P_minSDHFPAT_	6.1^‡^	13^‡^	-13^‡^	-0.8	-9.8^‡^	-0.2	-9.1^‡^	-1.7	-7.3^‡^
***σ***_***PAT***_	A B	P_rough_	P_peak_	P_foot_	P_pfd_	P_psd_	P_tanh_	P_tan_	P_maxagr_	P_minSDPAT_
	P_peak_	-6.8^‡^								
	P_foot_	8.2^‡^	10^‡^							
	P_pfd_	8.2^‡^	10^‡^	-5.6^‡^						
	P_psd_	9.8^‡^	11^‡^	-0.1	7.1^‡^					
	P_tanh_	9.2^‡^	11^‡^	-4.1^‡^	4.9^‡^	-5.2^‡^				
	P_tan_	11^‡^	11^‡^	2.9^‡^	9.5^‡^	2.5^†^	7.9^‡^			
	P_maxagr_	9.4^‡^	12^‡^	0.4	6.1^‡^	0.0	5.3^‡^	-1.0		
	P_minSDPAT_	12^‡^	13^‡^	11^‡^	12^‡^	11^‡^	12^‡^	10^‡^	12^‡^	
	P_minSDHFPAT_	7.6^‡^	10^‡^	-0.2	4.9^‡^	-0.8	3.5^‡^	-1.9	-4.4^‡^	-12^‡^
***ResprPAT***	A B	P_rough_	P_peak_	P_foot_	P_pfd_	P_psd_	P_tanh_	P_tan_	P_maxagr_	P_minSDPAT_
	P_peak_	5.0^‡^								
	P_foot_	3.1^†^	-1.4							
	P_pfd_	6.2^‡^	-1.0	0.5						
	P_psd_	5.5^‡^	-0.8	0.8	0.1					
	P_tanh_	7.0^‡^	0.0	3.3^‡^	5.9^‡^	2.2^†^				
	P_tan_	5.9^‡^	-1.0	3.1^†^	-0.2	0.2	-2.9^†^			
	P_maxagr_	9.8^‡^	2.3^†^	7.4^‡^	8.8^‡^	6.1^‡^	8.0^‡^	5.9^‡^		
	P_minSDPAT_	8.9^‡^	1.4	7.6^‡^	8.6^‡^	6.8^‡^	4.9^‡^	6.7^‡^	-2.3^†^	
	P_minSDHFPAT_	10^‡^	4.6^‡^	8.0^‡^	9.2^‡^	6.4^‡^	8.0^‡^	7.3^‡^	3.6^‡^	3.5^‡^
***σ***_***RR-IBI***_	A B	P_rough_	P_peak_	P_foot_	P_pfd_	P_psd_	P_tanh_	P_tan_	P_maxagr_	P_minSDPAT_
	P_peak_	-3.3^‡^								
	P_foot_	2.3^†^	4.4^‡^							
	P_pfd_	7.6^‡^	6.2^‡^	3.1^†^						
	P_psd_	6.4^‡^	6.1^‡^	1.4	-1.4					
	P_tanh_	9.8^‡^	7.9^‡^	7.9^‡^	8.5^‡^	5.2^‡^				
	P_tan_	9.1^‡^	8.3^‡^	11^‡^	5.8^‡^	6.7^‡^	-2.5^†^			
	P_maxagr_	12^‡^	13^‡^	12^‡^	12^‡^	11^‡^	9.1^‡^	8.2^‡^		
	P_minSDPAT_	11^‡^	8.2^‡^	8.8^‡^	5.8^‡^	6.2^‡^	2.3^†^	2.0^†^	-11^‡^	
	P_minSDHFPAT_	12^‡^	11^‡^	11^‡^	10^‡^	11^‡^	7.6^‡^	7.1^‡^	-10^‡^	8.2^‡^

## Discussion

This work has aimed to provide a new versatile tool for determining the arrival of pulse waves. We propose three different optimization criteria to obtain fiducial points, define the *PAT* time series (and, accordingly, the *IBI* time series), and compare the results with other fiducial points that can be found in the literature. Of course, other optimization criteria can be provided to optimize some kind of feature to suit the researcher’s needs. For example, if two pulse waves (one downstream of the other) are used instead of using the ECG signal and the pulse wave at a distal site, a feasible optimization criterion will be to minimize the standard deviation of the differentiation of the successive differences in pulse arrivals. Using this procedure (which implies optimizing $PA_{tag_{opt}}^{\alpha_{opt}}$for the proximal and distal pulse waves), a smooth *PTT* time series will be obtained that suits the hypothesis that changes in pulse wave velocity cannot have high-frequency contents because they are mediated by the sympathetic nervous system. In the remainder of this section, we will discuss only the results and their implications for the three employed optimization criteria.

The results in Table 1 and associated tests show that the values of *α*_*opt*_ and *tag*_*opt*_ are dependent on the choice of the optimization criteria, although *maxagr* and *minSDHFPAT* behave quite similarly with a high probability. The pulse wave is fractionally integrated (negative value of *α*_*opt*_), and the fiducial point then corresponds to the minimum during the systolic rise. Both criteria provide similar results because *maxagr* minimizes the standard deviation of the differentiated *PAT* time series, as shown in Eq (6), while *minSDHFPAT* minimizes the standard deviation of the fast (high-frequency band) changes in the resampled *PAT* time series. Both criteria aim to reduce similar factors. In contrast, the *minSDPAT* aims to minimize the standard deviation of any change in the *PAT* time series regardless of the spectral content of these changes. This criterion has been proposed for comparison with the performance of traditional fiducial points, especially when P_tanh_ claims to provide a minimum standard deviation of the *PAT* time series when compared with other traditional fiducial points. For *minSDPAT*, the optimal fiducial point can be obtained either by fractionally integrating or by fractionally differentiating the pulse wave. When using fractional integration, the chances that the fiducial point is the minimum are even greater than those for the other two optimization criteria. Moreover, when fractionally differentiating, the chances that the fiducial point corresponds to a maximum are also very high.

Table 2 shows that the values of *α*_*opt*_ and *tag*_*opt*_ depend mostly on the subject of interest. The posture has a mild influence on these values, while breathing has no statistical effect on them. The inter-subject variability is also an issue for the features of the *PAT* time series for all the analyzed fiducial points, as shown in Table 5. These changes among subjects can be explained by differences in the recorded pulse waveforms. Nevertheless, a change in waveform from one subject to another can be caused by changes in hemodynamics or changes in sensor attachment. Further research using a multi-sensor approach or deliberately changing the hemodynamics of the subject will be performed to better understand the role of the inter-subject variability in the values of *α*_*opt*,_*tag*_*opt*_ and different *PAT* features.

**Table 5:** Results of the Kruskal‒Wallis nonparametric test comparing the medians of several variables. The table shows the χ^2^ statistic and its significance († p<0.05, ‡ p<0.001). The factors subject (S), breathing (B) and posture (P) were tested separately. The tests are applied for the different fiducial points.

χ^2^		P_rough_	P_peak_	P_foot_	P_pfd_	P_psd_	P_tanh_	P_tan_	P_maxagr_	P_minSDPAT_	P_minSDHFPAT_
$\bar{PAT}$	S	107^‡^	92^‡^	127^‡^	129^‡^	143^‡^	121^‡^	142^‡^	82^‡^	68^‡^	74^‡^
	B	2.7	1.7	1.3	1.5	1.5	2.0	1.3	1.1	5.8	1.4
	P	11^‡^	35^‡^	1.0	6.6^†^	0.4	10^†^	1.3	5.9^†^	4.1	5.9^†^
*σ*_*PAT*_	S	80^‡^	66^‡^	75^‡^	80^‡^	89^‡^	83^‡^	94^‡^	82^‡^	81^‡^	84^‡^
	B	8.7^†^	1.9	7.1^†^	7.2^†^	7.6^†^	6.9^†^	8.2^†^	3.1	4.6	1.7
	P	1.0	1.4	5.5^†^	0.0	0.7	0.1	1.7	0.8	1.3	0.1
*ResprPAT*	S	98^‡^	36^†^	114^‡^	100^‡^	111^‡^	104^‡^	113^‡^	82^‡^	107^‡^	72^‡^
	B	20^‡^	19^‡^	14^‡^	24^‡^	12^†^	23^‡^	15^‡^	26^‡^	11^†^	34^‡^
	P	0.1	0.8	0.1	0.6	0.8	0.1	0.4	2.8	0.0	2.3
*σ*_*RR-IBI*_	S	125^‡^	82^‡^	129^‡^	108^‡^	130^‡^	128^‡^	136^‡^	125^‡^	120^‡^	123^‡^
	B	0.6	1.1	2.6	3.3	3.4	2.4	3.7	0.5	3.1	0.9
	P	0.5	2.6	1.2	4.5^†^	1.4	1.3	0.1	0.1	0.3	0.0

The first column in Table 3 shows that the mean value of *PAT* depends, of course, on the fiducial point choice. According to our database, the median value of this feature reaches a minimum for P_foot_, which is normal because it defines the start of the systolic increase and has a maximum for P_peak_, which marks the end of the systolic increase. In between and for increasing values of the mean of the *PAT*, we have P_psd_, P_tan_, P_minSDPAT_, P_maxagr_, P_pfd_, P_tanh_, P_minSDHFPAT,_ and P_rough_. Therefore, the large change in $\bar{PAT}$ associated with the fiducial point limits comparisons of results among studies with different fiducial point selections. On the other hand, the 10% to 90% percentile interval for $\bar{PAT}$ is greater for the fractionally differintegrated derived fiducial points than for the traditional fiducial points. The only two other fiducial points showing an interval greater than 100 ms are P_rough_ and P_peak_. Nevertheless, the causes of this high variability among these fiducial points obey different mechanisms. The final portion of the systolic increase slowly changes, where P_rough_ and P_peak_ lie. Any subtle change in morphology among recordings will explain the high variability in $\bar{PAT}$ when using P_peak_ or P_rough_. On the other hand, the high variability in $\bar{PAT}$ when using P_maxagr_, P_minSDPAT,_ or P_minSDHFPAT_ obeys the need to lie in some place between the start and the end of the systolic rise to suit the optimization criteria. Hence, the location is suitable for each recording. For some recordings and optimization criteria, the fractional differintegrated derived fiducial points can even reach the location of the foot or the peak of the systolic rise. The first block in Table 4 shows that, of course, P_peak_ provides a $\bar{PAT}$ with a median value very significantly greater than the rest of the fiducial points, while P_foot_ provides a value very significantly lower than the rest of the fiducial points. The only fiducial points where there were no significant differences when comparing the median $\bar{PAT}$ using P_pfd_ or P_maxagr_, when using P_tan_ or P_minSDPAT,_ and when using P_minSDHFPAT_ or either P_pfd_, P_tanh_ or P_maxagr_. These test results suggest that the most likely locations of P_maxagr_ and P_minSDHFPAT_ are near P_pfd_ and P_tanh_ respectively, close to the midpoint of the systolic rise, while P_minSDPAT_ is most likely located in the initial portion of the systolic rise, close to P_tan_. Nevertheless, the actual location depends on the recording. To finish the discussion on $\bar{PAT}$, the first block in Table 5 shows that the most influencing factor on this feature is the subject under measurement, regardless of the employed fiducial point. The posture significantly affects the feature of fiducial points located near the midpoint of the fiducial point (P_pfd_, P_tanh,_ P_maxagr,_ and P_minSDHFPAT_) and very significantly affects the feature of fiducial points located near the end of the systolic rise (P_rough_ and P_peak_). Morphological changes due to hydrostatic pressure changes and the physiological response to compensate for these changes may explain this effect. Nevertheless, further experimentation is needed to confirm whether these changes are associated with posture.

Table 3 shows the results for the median and the 10th to 90th percentiles of *σ*_*PAT*_ for the different fiducial points, while the second block of Table 4 shows the results for testing for significant differences in the median values of the paired samples. As expected by its optimization criterion, the fiducial point that provides the *PAT* time series with the lowest standard deviation is P_minSDPAT_, which shows very significant differences from the rest of the fiducial points. To increase the median value of *σ*_*PAT*_ come P_tan_, P_maxagr,_ P_minSDHFPAT_, P_foot_, P_psd_, P_tanh_, P_pfd,_ P_rough_ and, as expected, P_peak_ because of its sensitivity to noise. The performance of P_tanh_ is quite deceiving because Solà J et al. [26] claimed that it is a fiducial point that provides a low standard deviation *PAT*. These differences may be attributable to the differences in the recording sampling frequencies and experimental setup used to test the performance. Once again, *σ*_*PAT*_ is mostly influenced by the measured subject so it can be employed as a physiological index characterizing them. In contrast, posture seems not to affect value, and the effect of breathing can be significant depending on the employed fiducial point. *σ*_*PAT*_, defined by fractional differintegrated derived fiducial points, seems not to be affected by breathing or P_peak_. Although for P_peak_ this result can be attributed to the dominant noise effect in *σ*_*PAT*_, for P_maxagr_, P_minSDPAT_, and P_minSDHFPAT_, we hypothesize that this insensitivity to breathing stems from the ability of the algorithm to find fiducial points less affected by morphological changes associated with breathing. Nevertheless, further analysis of optimally fractional differintegrated pulse waves is needed to test this hypothesis. However, the effect of breathing on the rest of the fiducial points was significant but not as significant as the effect on the inter-subject variability.

For *ResprPAT*, the results follow a similar pattern as for *σ*_*PAT*_. Table 3 shows that breathing is a high-frequency process, and the lowest *ResprPAT* corresponds to P_minSDHFPAT_. Next, they included P_maxagr_, P_minSDPAT_, P_tan_, P_psd_, P_tanh_, P_peak_, P_pfd_, P_foot_ and P_rough_. The most sensitive traditional fiducial points for breathing are most likely located in the middle to last section of the systolic rise. The *PAT*, when using the P_foot_ test, also has a greater modulation associated with breathing. The third section of Table 4 shows that *ResprPAT* is very significantly lower for P_minSDHFPAT_ than for the other studied fiducial points, as predicted by the employed optimization criterion. Inter-subject variability is the factor that most affects the *ResprPAT*, while posture seems not to influence its value. Depending on the fiducial point, breathing can have a very significant influence or a mildly significant influence (this is the case for P_minSDPAT_ and P_psd_). These influences suggest that the variability in the *PAT* associated with breathing is dependent on the respiratory rate.

For the agreement between the *RR* and the *IBI* time series, the quantifier *σ*_*RR*-*IBI*_ is, as foreseeable, the minimum when using P_maxagr_ as shown in Table 3. The fourth block in Table 4 also shows that this fiducial point shows very significant differences, with the rest of the fiducial points being *σ*_*RR*-*IBI*_ consistently lower than when using other alternatives. After P_maxagr_, the best agreement is obtained for P_minSDHFPAT_, followed by P_minSDPAT_ and P_tan_. Note that for optimization purposes, the fractional differintegration derived fiducial points require knowledge of the R wave location to define the fiducial point location although the remaining fiducial points do not need to define the *IBI* time series. Hence, P_tan_ is, using our database, the best fiducial point for defining a time series with likely good agreement with the *RR* time series without the need to know the R peak locations. After P_tan_, sorted by increasing *σ*_*RR*-*IBI*_, come to P_tanh_, P_foot_, P_psd_, P_pfd_, P_rough_, and, finally and not surprisingly, P_peak_. Table 5 shows that inter-subject variability is the most influencing factor on agreement, while breathing and posture have small effects. As is the case for *σ*_*PAT*_,*σ*_*RR*-*IBI*_ can be used as a physiological marker for the subjects being measured.

In HRV measurements, two time domain indices are commonly used in the literature [38]: the SDNN, which corresponds to the standard derivation of the *RR* time series, and the rmsDD, which corresponds to the standard deviation of the first differentiation of the *RR* time series. Note that *σ*_*PAT*_ is the same index as SDNN but is applied to the *PAT* time series, while *σ*_*RR-IBI*_ is similar to rmsDD for the *PAT* time series in accordance with Eq (6). Nevertheless, although the different indices in HRV analysis are slightly affected by the fiducial point employed to define the R wave (because of its impulsive nature), the analysis of *PAT* variability is strongly influenced by the fiducial point choice, as the measured values of $\bar{PAT}$,*σ*_*PAT*_ *ResprPAT*, and *σ*_*RR-IBI*_ can significantly change among fiducial points.

As shown in the results, all the analyzed features and optimization procedures depend on the subject under measurement (and probably how the sensors are attached). Hence, our results can change by expanding the database of interest. Future work will include other physiological databases that include simultaneous measurements of ECG and PPG signals to test how the results change for different experimental setups (e.g., PPG location, subject posture, sampling frequency, and movement restrictions). Although, for the setup of our measurements, the number of outliers was kept low, the results change slightly by modifying the outlier detection criterion. It is foreseeable that the results would change for other databases with a large number of outliers. Analysis of new databases could also serve as a way to test whether the proposed new set of fiducial points is robust to noisy events such as movement or poor sensor attachment.

Finally, the *PAT* has been proposed as a surrogate measure of *PTT* that is strongly correlated with blood pressure. As indicated in the introductory section, some studies acknowledge the utility of *PAT* for noninvasively estimating blood pressure, but others indicate that these blood pressure estimations are not accurate enough. These differences in accuracy may be caused by the choice of fiducial point. These studies focused on estimations of the average value of blood pressure (both systolic and diastolic) from the mean values of either the *PAT* or *PTT*. We hope that beat-to-beat analysis of *PAT* could provide a better way to noninvasively track the dynamics of blood pressure if the right fiducial point is selected. Because the regulation of pulse wave velocity and blood pressure is dominated by low-frequency processes, we hypothesize that optimization criteria such as *minSDHFPAT* or *maxagr*, which try to minimize the high-frequency of *PAT*, may work better in the quest for beat-to-beat blood pressure estimation. Hence, further experimentation with direct measurements of beat-to-beat blood pressure or beat-to-beat pulse wave velocity could refute or compromise the utility of this new set of fiducial points.

## Conclusions

This paper has proposed a new set of fiducial points based on the fractional differintegration of pulse waves to improve the detection of pulse arrivals. As the set of fiducial points is infinite, several optimization criteria can be proposed to improve detection based on different needs. This paper has proposed three optimization criteria to maximize the agreement of the *IBI* time series with the *RR* time series, to minimize the standard deviation of the *PAT* time series, or to minimize the standard deviation of the high-frequency contents of the *PAT* time series. To assess the performance of the proposed method, this study investigated seven traditional fiducial points commonly used in *PAT* and *IBI* and compared their performance against a newly proposed set of fiducial points. The chosen characteristics for evaluating the fiducial points included accurate pulse arrival detection, consistency with the *RR* time series, and robustness to variability. The best-performing fiducial points showed accurate pulse arrival detection, alignment with the *RR* time series, and robustness to variations in pulse wave morphology. In contrast, the worst-performing fiducial points exhibit poor adaptability to physiological changes, including posture changes, inaccurate pulse arrival detection, and vulnerability to external factors such as breathing patterns. The results showed that our proposal can provide an *IBI* time series with better agreement with the *RR* time series rather than other traditional fiducial points (median agreement of 5.72 ms in contrast with 6.62 ms for P_tanh_, 7.29 ms for P_foot_ or even 9.93 ms for P_peak_); a *PAT* series with a lower standard deviation than other traditional methods (median standard deviation of 7.21 ms versus 8.67 ms for P_tanh_, 8.22 ms for P_foot_ or 15.4 ms for P_peak_); and a *PAT* series with lower respiratory modulation than other fiducial points (median respiratory contribution of 3.44 ms versus 4.50 ms for P_tanh_, 4.72 ms for P_foot_ or 4.56 ms for P_peak_).

According to this result, the proposed fiducial points showed a notable reduction in the standard deviation of the *PAT* compared to traditional points, suggesting its potential for a more accurate assessment of beat-to-beat *PAT* variability and *IBI* time series.

Further research may define better optimization criteria to improve the measurement of beat-to-beat *PAT* variability as a surrogate measurement of the dynamics of the pulse wave velocity (*PWV*).
